# Low-dose rituximab should be used for treating MS in resource-limited settings: No

**DOI:** 10.1177/13524585221089889

**Published:** 2022-04-18

**Authors:** Farrah J Mateen

**Affiliations:** Department of Neurology, Massachusetts General Hospital, Boston, MA, USA

While patients with multiple sclerosis (MS) in most wealthy settings have access to more than 20 disease-modifying therapies (DMTs), patients in resource-limited settings often have access to none. This disparity in MS treatment access is extreme compared to the many diseases—neurological and otherwise—for which effective, scientifically proven treatments exist.

Many countries worldwide procure drugs according to the World Health Organization’s *Model List of Essential Medicines*.^1^ Since there are no MS DMTs listed, immunosuppressive agents, including rituximab and its quality-assured biosimilars, listed for other indications, should be considered for the treatment of MS. Among low-income countries that procure a limited supply of “on-label” MS DMTs, the most common are interferon beta and glatiramer acetate.^2^ Patients with MS with only these two options may alternate between low-efficacy agents each time they have a disease attack. This unfortunate situation requires renewed thinking on how best to treat people with MS in resource-limited settings.

There are practical advantages to rituximab use for MS in resource-limited settings, including its appropriate use in multiple mimicking central nervous system (CNS) demyelinating disorders such as neuromyelitis optica. Semi-annual dosing of a DMT is pragmatic for highly mobile resource-limited populations, such as refugees.^3^ While these pragmatic factors justify B-cell therapies as a treatment approach in general, they do not justify low-dosing. By contrast, barriers to intravenous rituximab treatment are few infusion centers, usually concentrated in large cities; scarcity of skilled personnel to treat MS; and the increased need for laboratory screening and monitoring compared to other DMTs.

People in resource-limited settings with MS—like all people with MS—deserve highly effective, safe, tolerable, and affordable treatments that have a robust evidence base. The question of whether *low-dose* rituximab is as efficacious, safer, and more affordable should be examined for people with MS.

Knowledge on the efficacy of low-dose rituximab is inadequate for people with MS to recommend it routinely as a standard of care. Importantly, no standard definition of low-dose exists, and no consensus dosing strategy of rituximab in neuroinflammatory disease has yet transpired.^4^ Initial definitions of low dose range from 100 mg to 1000 mg per dose or cycle.^5–8^ An observational cohort from Sweden found that patients treated with a median dose of 500 mg intravenous (IV) every 6 months was highly effective in some people with MS.^5^ However, there are few long-term follow-up studies of low-dose rituximab including disability outcomes.

In all settings, rational dosing of rituximab is needed, including a focus on the therapeutic goal, which is the degree of B-cell depletion, not the overall dose.^4^ It is uncertain whether dosing studies of rituximab in MS in high-income settings^6^ extrapolate well to patients in resource-limited settings—who may be on average younger, of lower body mass index, and have different disease risk factors. While real-world data from low-income settings is emerging and low-dose rituximab appears promising, the picture remains incomplete. When a tiered dosing strategy of rituximab was tested in 118 people with MS in India, the authors found dosing of 500 mg IV rituximab every 9–12 months in 34 patients appeared effective.^7^ Low-dosing was administered to patients with less disease activity via pre-treatment disease assessment of patients with magnetic resonance imaging (MRI) and follow-up evaluation clinically alongside serial flow cytometry for serum B-cell subsets. By contrast, a pre-print study^8^ of 85 Iranian people with MS treated with rituximab (500 mg 2 weeks apart every 6 months) reported 18 patients experienced a relapse over a 4-year observation period. Notably, the initiation of rituximab in these patients was based on a poor clinical response to a first-line DMT. Financial constraints precluded the use of flow cytometry in all patients.

There remains equipoise on which patients would benefit from low-dose rituximab versus higher-dosed rituximab. Prospective, randomized studies comparing doses are required in resource-limited settings, including in children. While many people with MS would likely benefit more from low-dose rituximab compared to interferons or glatiramer acetate, these same patients would likely benefit even more from higher-dosed B-cell therapies. Selection of the subset of MS patients who could be effectively treated with the lowest doses may require additional expenditures in laboratory tests, neuroimaging, and personnel, expenditures that could obviate the costs of lower dosing.

Beyond efficacy, there are two main arguments for the preferential use of low-dose to high-dose rituximab in resource-limited settings: (1) lower risk of serious infections and hypogammaglobulinemia and (2) lower cost.

The risks of infection during immunosuppression could be higher in resource-limited settings for a variety of reasons, including the wide range of pathogens found in tropical zones and the impact of poverty on infectious diseases. However, limited data substantiate the hypothesis that low-dose rituximab leads to lower rates of serious infection in people with MS compared to typically used higher doses.^5^ Serious infections have not been disproportionately reported in MS patients treated with rituximab in resource-limited settings.^6–9^

The second main argument to lower dose is cost. Assuming an average cost of 23USD per 10 mL vial of rituximab (10 mg/mL) in resource-limited settings,^10^ a dose of 1000 mg would cost ~2,300USD per cycle or 4,600USD annually if dosed every 6 months. Since many patients in resource-limited settings pay for medicines out of pocket, only ultra-low-dose rituximab (<500 mg every 6 months) could be affordable without additional financial support. The World Bank estimates 40 countries have a gross national income (GNI) per capita of <1500USD per year. Notably, these costs do not include laboratory screening, administration by skilled health care workers, additional medicines to improve tolerability, or patients’ transportation costs. Moreover, since MS affects disproportionately young women, a demographic group already disadvantaged in several resource-limited settings in terms of education, employment opportunity, personal income, and social safety nets, cost almost certainly remains a major determinative factor in treatment choice for most people living with MS in the poorest settings.

When cost of an effective drug is the most critical factor in making a dosing determination for a disabling and life-threatening disease, more must be done by the global community than recommend a lower dose. One must question whether lower doses of any effective drug can be ethically recommended based on cost alone in MS. In other diseases, including HIV/AIDS, insulin-dependent diabetes, and chronic myeloid leukemia, re-negotiation of drug pricing and ensuring adequate supply chains of life-sustaining treatments have occurred. Although not straightforward, political will, advocacy, and science have come together to improve cost. People with MS in resource-limited settings should not be subject to no treatment, less-adequate treatment, less robust data for their DMT use, or fewer treatment options due to issues of cost alone. The MS field can achieve rational drug pricing and evidence-based drug dosing for patients in resource-limited settings.

## References

[1] World Health Organization model list of essential medicines – 22nd list. geneva: World Health Organization, 2021.

[2] Jamal I Shah J Mativo P , et al. Multiple sclerosis in Kenya: Demographic and clinical characteristics of a registry cohort. Mult Scler J Exp Transl Clin 2021; 7(2): 1–10. 10.1177/20552173211022782 PMC8209835 34188951

[3] Zeineddine MM Yamout BI . Treatment of multiple sclerosis in special populations: The case of refugees. Mult Scler J Exp Transl Clin 2020; 6(1): 1–7. 10.1177/2055217319848466 PMC6956602 31976080

[4] Whittam DH Tallantyre EC Jolles S , et al. Rituximab in neurological disease: Principles, evidence and practice. Pract Neurol 2018; 19: 5–20. 30498056 10.1136/practneurol-2018-001899

[5] Salzer J Svenningsson R Alping P , et al. Rituximab in multiple sclerosis: A retrospective observational study on safety and efficacy. Neurology 2016; 87: 2074–2081. 27760868 10.1212/WNL.0000000000003331 PMC5109942

[6] Nielsen AS Miravalle A Langer-Gould A , et al. Maximally tolerated versus minimally effective dose: The case of rituximab in multiple sclerosis. Mult Scler 2012; 18(3): 377–378. 21828201 10.1177/1352458511418631

[7] Mathew T John SK Kamath V , et al. Efficacy and safety of rituximab in multiple sclerosis: Experience from a developing country. Mult Scler Relat Disord 2020; 43: 102210. 32485634 10.1016/j.msard.2020.102210

[8] Mokhtari M Abolmaali M Emamikhah M , et al. Effectiveness and safety of low-dose Rituximab in the treatment of demyelinating diseases of the central nervous system. Mult Scler Relat Disord 2020; 43: 102210.32485634

[9] Bribiesca-Contreras E García-Estrada C Gómez-Figueroa E , et al. Impact of rituximab in Mexican patients with multiple sclerosis—A single-center retrospective study. Mult Scler Relat Disord 2022; 58: 103485. 35042092 10.1016/j.msard.2021.103485

[10] Management Sciences for Health (MSH). International medical products price guide. 2015 ed. (Updated annually). Medford, MA: MSH, 2016.

